# An Unusual Presentation of Severe Sepsis Due to Clostridium difficile Enteritis

**DOI:** 10.7759/cureus.4162

**Published:** 2019-03-01

**Authors:** Haisam Abid, Edward Bischof

**Affiliations:** 1 Internal Medicine, Bassett Medical Center, Cooperstown, USA

**Keywords:** c. difficle, enteritis

## Abstract

We report an atypical case of Clostridium difficile (C.difficile) infection in a 35-year male who presented to the hospital because of diffuse abdominal pain associated with nausea and vomiting. Patient denied diarrhea or hematochezia. On physical examination, he was afebrile, but tachycardic and hypotensive. Abdominal examination revealed mild diffuse tenderness without signs of peritonitis. Lab work up was significant for leucocytosis and elevated serum lactate. Computed tomography (CT) of the abdomen and pelvis with intravenous (IV) contrast was done with findings suggestive of enteritis. Initial work up did not reveal any source of infection, so he was treated with broad-spectrum antibiotics for severe sepsis of unknown origin. Broad-spectrum antibiotics were continued for two days without significant improvement in signs and symptoms; stool studies were obtained which showed positive C.difficile on polymerase chain reaction (PCR) after which oral vancomycin was started and IV antibiotics were stopped. The patient's signs and symptoms improved after a couple of days of oral vancomycin and he was discharged home to complete a 14-day course of oral vancomycin.

## Introduction

The most common symptom of Clostridium difficile (C.difficile) infection is watery diarrhea and is generally a disease of the colon [1]. However, involvement of the small bowel is very rare and is associated with a high mortality rate of 23%-69% due to delay in diagnosis [2]. Establishing the correct diagnosis of C.difficile enteritis is challenging because in addition to the rarity of the infection involving the small bowel, radiological manifestations of C.difficile associated enteritis are not as well understood as the imaging appearance of colitis [1-2].

## Case presentation

**Figure 1:**
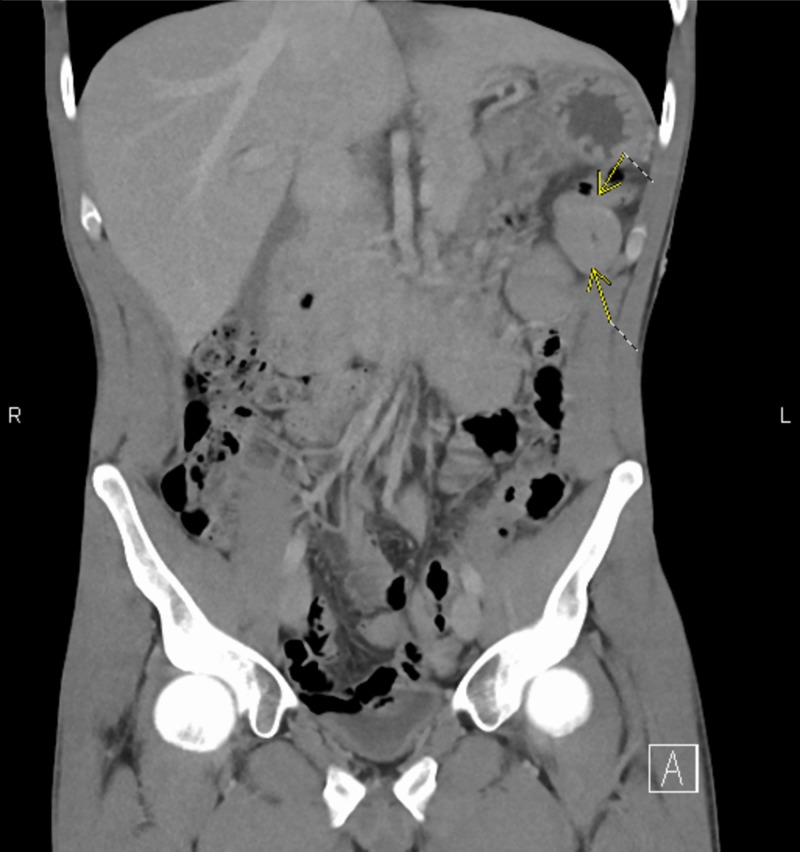
Coronal computed tomography (CT) of the abdomen and pelvis with intravenous contrast showing mild-moderate wall thickening of the jejunum

A 35-year-old man with a medical history of splenectomy due to splenic artery rupture presented to the hospital with diffuse abdominal pain of one-day duration associated with nausea and two episodes of non-bilious, non-bloody emesis. Patient denied any significant aggravating or relieving factors of the pain, no association with food intake or recent antibiotic exposure, and no fever, chills, rigors or diarrhea. He was not taking proton pump inhibitor. On examination, the patient was afebrile, tachycardic with a blood pressure of 85/61 mm Hg. Abdominal examination revealed diffuse mild tenderness without guarding or rigidity and bowel sounds were present. Lab work up was pertinent for leucocyte count of 32 x 109 cells/L (normal range: 3.7-11 x 109 cells/L) with predominant neutrophils 87% and elevated serum lactate 4 mmol/L (normal range: 0.5-1.0 mmol/L) with no end-organ damage. Urine, blood cultures, and chest X-ray did not reveal any source of infection. Computed tomography (CT) of the abdomen and pelvis with intravenous (IV) contrast showed mild-moderate prominence of adjacent proximal and mid jejunum without bowel obstruction or evidence of colitis, most likely representing enteritis (Figure 1). The patient was started on broad-spectrum antibiotics with IV pipercillin-tazobactam and vancomycin due to concern for severe sepsis of unclear etiology. As patient signs and symptoms did not improve with broad-spectrum antibiotics, stool studies were obtained and C.difficile was confirmed on stool polymerase chain reaction (PCR). The patient was started on oral vancomycin 125 mg every six hours and IV antibiotics were discontinued. The patient’s signs and symptoms improved after oral vancomycin, and he was discharged home to complete a 14-day course of oral vancomycin.

## Discussion

C.difficile is an anaerobic motile gram-positive bacillus that is spread by bacterial spores found within feces. With widespread use of antibiotics in the healthcare system, the incidence of C.difficile infection is increasing. The most common symptom of C.difficile infection is watery diarrhea [3]. Other symptoms which can be seen with C.difficile infection are abdominal pain, fever, nausea, vomiting, and sometimes blood in the stool.

The most common risk factors associated with C.difficile infection are recent antibiotic use, immunosuppression, previous bowel surgery especially colectomy or ileostomy, and inflammatory bowel disease [4-5]. Out of all these risk factors, colectomy is considered the most common risk factor leading to C.difficile enteritis as it alters the small bowel flora by disturbing normal small bowel peristalsis and normal functioning of the ileocecal valve [6]. However, our patient did not have any of these risk factors which is a very rare finding.

Given the rarity of enteritis, its radiological manifestations are not well documented. Our patient only had mucosal thickening of the mid and proximal jejunum. The differential diagnosis of this finding is broad: infection, auto-immune disorder, ischemia or vasculitis. Therefore, clinical correlation is necessary to narrow down to the correct diagnosis.

## Conclusions

Our patient presented with an unusual form of C.difficile involving only the small bowel. Several typical features and risk factors for C.difficile infection were not present. This case emphasizes the importance of considering C.difficile in the differential diagnosis of enteritis.

## References

[1] Khan SA, Towheed A, Llah ST, Abdulhak AB, Tilson-Mallett NR, Salkind A (2016). Atypical presentation of C. Difficile infection: report of a case with literature review. Cureus.

[2] Siddiqui J, Campion T, Wei R, Kuzmich S (2018). Clostridium difficile enteritis: diffuse small bowel radiological changes in a patient with abdominal sepsis. BMJ Case Rep.

[3] Dineen SP, Bailey SH, Pham TH, Huerta S (2013). Clostridium difficile enteritis: a report of two cases and systematic literature review. World J Gastrointest Surg.

[4] Wee B, Poels JA, McCafferty IJ, Taniere P, Olliff J (2009). A description of CT features of Clostridium difficile infection of the small bowel in four patients and a review of literature. Br J Radiol.

[5] Lavallée C, Laufer B, Pepin J, Mitchell A, Dubé S, Labbé AC (2009). Fatal Clostridium difficile enteritis caused by the BI/NAP1/027 strain: a case series of ileal C. difficile infections. Clin Microbiol Infect.

[6] Hayetian FD, Read TE, Brozovich M, Garvin RP, Caushaj PF (2006). Ileal perforation secondary to Clostridium difficile enteritis: report of 2 cases. Arch Surg.

